# Supernumerary B Chromosomes and Plant Genome Changes: A Snapshot of Wild Populations of *Aegilops speltoides* Tausch (*Poaceae*, Triticeae)

**DOI:** 10.3390/ijms21113768

**Published:** 2020-05-26

**Authors:** Imad Shams, Olga Raskina

**Affiliations:** Institute of Evolution and Department of Evolutionary and Environmental Biology, University of Haifa, Haifa 3498838, Israel; imadshams@gmail.com

**Keywords:** *Aegilops speltoides*, B chromosome, ectopic recombination, genome evolution, LINE, retrotransposon copy numbers, somatic recombination, tandem repeats, Ty1-*copia*, Ty3-*gypsy*

## Abstract

In various eukaryotes, supernumerary B chromosomes (Bs) are an optional genomic component that affect their integrity and functioning. In the present study, the impact of Bs on the current changes in the genome of goatgrass, *Aegilops speltoides*, was addressed. Individual plants from contrasting populations with and without Bs were explored using fluorescence in situ hybridization. In parallel, abundances of the Ty1-*copia*, Ty3-*gypsy*, and LINE retrotransposons (TEs), and the species-specific Spelt1 tandem repeat (TR) in vegetative and generative spike tissues were estimated by real-time quantitative PCR. The results revealed: (i) ectopic associations between Bs and the regular A chromosomes, and (ii) cell-specific rearrangements of Bs in both mitosis and microgametogenesis. Further, the copy numbers of TEs and TR varied significantly between (iii) genotypes and (iv) different spike tissues in the same plant(s). Finally, (v) in plants with and without Bs from different populations, genomic abundances and/or copy number dynamics of TEs and TR were similar. These findings indicate that fluctuations in TE and TR copy numbers are associated with DNA damage and repair processes during cell proliferation and differentiation, and ectopic recombination is one of the mechanisms by which Bs play a role in genome changes.

## 1. Introduction

Numerous species of animals, plants, and fungi have supernumerary B chromosomes (Bs), which are known as a facultative genomic component. However, Bs are able to affect genome stability and functioning [1], [2]. The notable features of Bs are variability in numbers among individuals, species-specific organization, and enrichments with different types of repetitive DNA; and the inheritance of the B chromosome is not subject to Mendelian laws [1,2,3,4,5,6,7,8]. Generally, the presence of Bs, especially in high numbers, is associated with destabilization of the genome that, consequently, implies selection against “selfish” chromosomes. The origin of Bs is still obscure, but studies employing recent advances in next-generation sequencing indicate that they arise as a result of A chromosome rearrangements, and some genes of standard A chromosomes remain transcriptionally active in Bs [2,9,10]. In the present research, we focused on the impact of Bs on the current changes in the genome of goatgrass, *Aegilops speltoides* Tausch (SS-genome, 2n = 2x = 14; sect. *Sitopsis*), which is a wild progenitor of the B and G genomes of allopolyploid wheats [11,12]. *Ae. speltoides* is a dimorphic species, and the ssp. *ligustica* (dominant) and ssp. *aucheri* (recessive) morphotypes co-exist at varying ratios in wild populations [13,14]. This is a predominantly cross-pollinated but self-compatible species, which under stressful conditions transits to self-pollination [13,14]. 

In the wild panmictic populations of *Ae. speltoides*, Bs vary in numbers between individuals and stably present in the aerial organs in all cells, but they are usually absent in the roots, where elimination occurs in the early stages of development [15,16]. The transmission of Bs is accompanied by nondisjunction of sister chromatids and unequal spindle organization during the first pollen grain mitosis [15,16]. Further, Bs accumulate primarily in the generative nuclei and are very rare in the vegetative nuclei [15,16]. One of the consequences of various types of stress throughout the species distribution area is an increase in the frequency and canalization of chromosomal aberrations [17]. Current chromosome rearrangements, which have been fixed in populations, cause intraspecific diversification in repetitive DNA patterns of standard A chromosomes, and this is also true for Bs. In *Ae. speltoides*, Bs are significantly shorter than regular A chromosomes and differ from them in repetitive DNA patterns. A distinguishing and well-conserved feature of Bs is the presence of 5S rDNA loci in both chromosome arms; in the long arm, the small Spelt1 cluster is located proximal to the 5S rDNA cluster [17,18,19,20]. Proximal to the 5S rDNA and Spelt1 clusters in the long arm, the Ty1-copia [21] and Ty3-gypsy [22] retrotransposons constitute large intercalary blocks. Furthermore, in *Ae. speltoides*, Bs are highly enriched in chloroplast- and mitochondria-derived DNA [19] and recently identified specific repeats [16]. Highly enriched in repetitive sequences, which are the hot points for chromosomal rearrangements, Bs demonstrate polymorphism in their repetitive DNA patterns and morphology [16,17,19].

In this study, individual plants from contrasting populations with and without Bs were explored by means of fluorescence in situ hybridization (FISH) technique. Cytogenetic analysis revealed a wide spectrum of ectopic associations between B and A chromosomes and rearrangements of Bs during both mitosis and microgametogenesis. Along with cytogenetic analysis, the abundances of the Ty1-*copia*, Ty3-*gypsy*, and LINE retrotransposons (TEs) and the species-specific Spelt1 tandem repeat (TR) in vegetative and generative spike tissues were estimated by real-time quantitative PCR (qPCR). We found that the copy numbers of TEs and TR vary significantly between genotypes and between different spike tissues within individual plants. An unexpected finding was that in plants with and without Bs from different populations, the TE and TR abundances and/or copy number dynamics were similar. Altogether, the data obtained indicate that fluctuations in TE and TR copy numbers are associated with DNA damage and repair processes during cell proliferation and differentiation, and ectopic recombination is one of the mechanisms by which Bs are involved in genome changes. These findings imply that when supernumerary chromosomes are present in small numbers, they do not adversely affect the genome but, rather, provide advantages that are useful under stressful or fluctuating environmental conditions. This advantage explains the maintenance of Bs in natural populations. 

## 2. Results

In the present study, rearrangements of Bs and regular A chromosomes and inter-chromosomal associations in somatic and meiotic cells were revealed. Repetitive sequences are the main targets for recombination; therefore, the evolutionarily conserved 5S rDNA, Spelt1, CCS1, and (TTTAGGG)*_n_* telomere repeats were utilized specifically as markers for B chromosome rearrangements.

### 2.1. Rearrangements of B Chromosomes and Ectopic Associations with Standard A Chromosomes During Mitosis and Meiosis

#### 2.1.1. Ectopic B-A Chromosome Associations and Rearrangements of B Chromosomes in Somatic Cells

**Figure 1:**
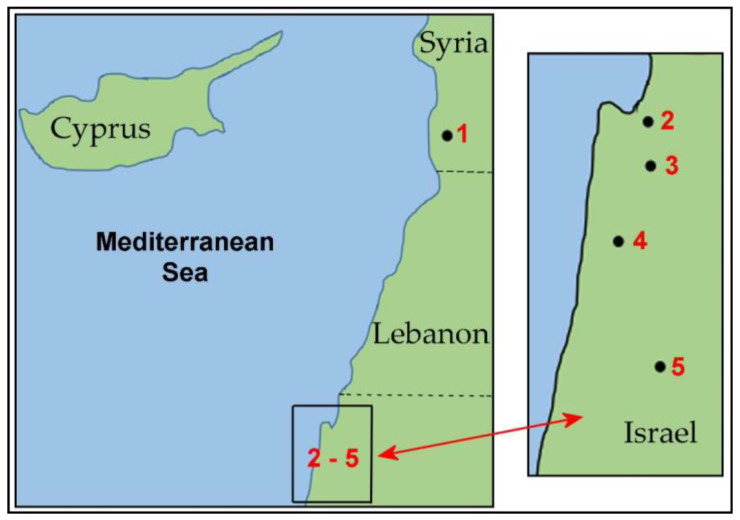
Geographical location of the studied populations of *Ae. speltoides*. Collection sites for *Ae. speltoides*: 1—Tartus; 2—Kishon; 3—Technion; 4—Ramat haNadiv; 5—Katzir.

In all the investigated plants (Figure 1; Table 1), which carry 1 to 6 Bs, ectopic associations between B and regular A chromosomes and rearrangements of Bs were revealed. Among 10 to 15 well-spread chromosome plates analyzed on the single cytological slide, in 2 to 5 cases, ectopic associations between B and standard A chromosomes and/ or B chromosome rearrangements were detected in a cell-specific manner. However, the real amount of cell-specific events was underestimated as a large number of cells were ignored because of a failure to accurately discriminate ectopic associations and chromosome overlapping.

**Figure 2:**
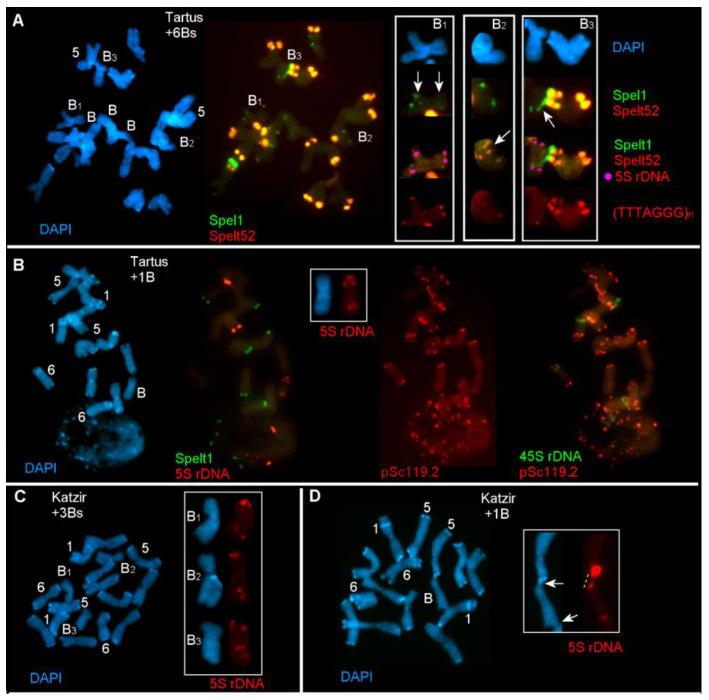

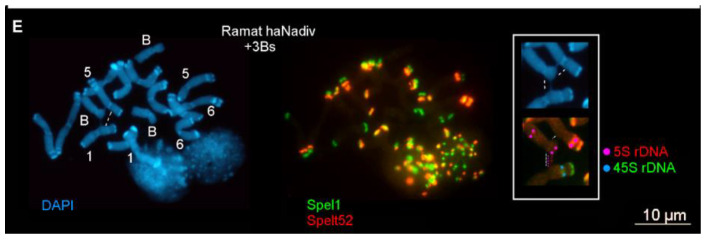
Rearrangements of B chromosomes and ectopic B-A chromosome associations in somatic cells in different genotypes of *Aegilops speltoides*. Fluorescence in situ hybridization (FISH) analysis was conducted on somatic chromosomes of plants from the populations of Tartus (**A**,**B**), Katzir (**C**,**D**), and Ramat haNadiv (**E**). DNA probes for FISH: Spelt1 (in green), Spelt52 (in red), pSc119.2 (in red), 5S rDNA (in red), 45S rDNA (in green), and (TTTAGGG)*_n_* (in red); differential staining with DAPI (in blue). Only chromosomes 1, 5, 6, and B are numbered. (**A**) Somatic chromosomes of the plant from Tartus (2*n* = 2x = 14 + 6B). A single pair of A chromosomes carries a large Spelt1 cluster. One B chromosome (B_1_) carries additional Spelt1 clusters in both arms, which is indicative of rearrangement (arrows, enlargement in the small box B_1_). Re-probing with 5S rDNA (in pink pseudo color; the second round of FISH) and (TTTAGGG)*_n_* (the third round of FISH) are shown for B_1_ chromosome in the small box B_1_. (TTTAGGG)*_n_* clusters are revealed in both arms. Another B chromosome (B_2_) carries a translocation in the short arm comprising the 5S rDNA and Spelt1 clusters (arrow, enlargement in the small box B_2_). A single (TTTAGGG)*_n_* cluster is revealed in one chromatid in the long arm. Ectopic B-A association through the Spelt1 fiber is revealed in this cell (arrow, enlargement in the small box B_3_). 5S rDNA clusters in B_3_ are indicated in pink pseudo color (the second round of FISH). (TTTAGGG)*_n_* clusters (the third round of FISH) are observed in the B_3_ long arm and deleted in the short arm. (**B**) Somatic chromosomes of the plant from Tartus (2*n* = 2x = 14 + B). FISH with Spelt1 (in green) and 5S rDNA (in red). Three pairs of A chromosomes carry large Spelt1 clusters. The B chromosome possesses two 5S rDNA clusters in the short arm (enlargement in the small box). Re-probing (the second round of FISH) with pSc119.2 (in red) and 45S rDNA (in green). No pSc119.2 clusters were found on the B chromosome. (**C**) Somatic chromosomes of the plant from Katzir (2*n* = 2x = 14 + 3B). One B chromosome (B_1_) demonstrates standard pattern of 5S rDNA clusters (B_1_, enlargement in the small box; top). In chromosome B_2_, two 5S rDNA clusters are located in the same chromatid in the long arm, indicating intrachromosomal rearrangement (B_2,_ enlargement in the small box; middle). An additional 5S rDNA cluster in the long arm of the third B chromosome (B_3_) is revealed (B_3_, enlargement in the small box; bottom). (**D**) Somatic chromosomes of the plant from Katzir (2*n* = 2x = 14 + B). Clusters of 5S rDNA in the short arms of chromosomes 5 and B are ectopically connected (arrow and dashed line, enlargement in the small box); the long arm of chromosome B is connected with the short arm of chromosome 1 (arrow, enlargement in the small box). (**E**) Somatic chromosomes of the plant from Ramat haNadiv (2*n* = 2x = 14 + 3B). FISH with Spelt1 (in green) and Spelt52 (in red); re-probing with 5S rDNA (in red) and 45S rDNA (in green). Ectopic associations between one B and two A chromosomes are stressed (dashed lines, enlargement in the small box). In the small box, 5S rDNA and 45S rDNA clusters are indicated in pink and blue pseudo color, respectively.

The data obtained show that different A chromosomes can be randomly involved in somatic associations with Bs. Somatic chromosome plate of the plant from the population of Tartus is shown in Figure 2A. This population is defined as marginal according to its eco-geographical characteristics and enrichment with the Spelt1 and Spelt52 TRs [14,17] (Figure 1; Table 1). There are six Bs in this genotype (2*n* = 2x = 14 + 6Bs). A single pair of A chromosomes carries a large Spelt1 cluster; chromosome 4 carries a small intercalary Spelt1 cluster; and all Bs carry a typical small Spelt1 cluster in the long arm. In this genotype, cell-specific rearrangements of B chromosomes were detected. Particularly, in one B chromosome (B_1_), (Figure 2A, small box B_1_), an atypical second Spelt1 cluster was revealed in the long arm, and an additional small Spelt1 cluster was discovered proximal to the 5S rDNA clusters in the short arm. The clusters of the telomere repeat (TTTAGGG)*_n_* were found in both arms. Another B chromosome (B_2_) is shown in the insert B_2_. Here, the short arm carries the translocated fragment comprising 5S rDNA and Spelt1 clusters, and this is, likely, a part of the long arm of another B. A single (TTTAGGG)*_n_* cluster was found in one chromatid in the long arm, while other telomere clusters, evidently, were deleted as a result of rearrangement(s). In the third insert B_3_ (Figure 2A), the focus is on ectopic B-A associations. Specifically, the fiber of the Spelt1 tandem repeat connects the A and B chromosomes. In the B short arm (B_3_), the telomere clusters are deleted. Hence, at least two of the six B chromosomes are rearranged, and a third B chromosome is involved in the ectopic B-A association in this somatic cell.

Somatic chromosomes of another plant from the population of Tartus are shown in Figure 2B. This plant possessed a single B (2*n* = 2x = 14 + B). In contrast to the previous genotype, three pairs of A chromosomes carried a large Spelt1 cluster. In the B short arm, an additional 5S rDNA cluster was observed in all the cells; therefore, this was a genotype-specific characteristic. The tandem repeat pSc119.2 is highly abundant in the *Ae. speltoides* genome and has been reported in Bs [18]. However, in this research and in a previous one [20], pSc119.2 was not found in Bs. In Figure 2C,D, cell-specific rearrangement and ectopic B–A associations are shown for two plants from the population of the intermediate type of Katzir [14,17] (Figure 1; Table 1). In one of the plants, two of the three Bs were rearranged (2*n* = 14 + 3B) (Figure 2C). In particular, in B_2_, both clusters of 5S rDNA in the long arm were found to be located in the same chromatid; this indicates somatic intrachromosomal recombination. The supernumerary B_3_ carried two 5S rDNA clusters in the long arm, and this also appears to be the result of somatic rearrangement. The second plant had a single B chromosome in the genome (2*n* = 14 + B); and somatic B-A associations are stressed in Figure 2D. Specifically, chromosome 5 and the short arm of the B chromosome were ectopically linked via an extended 5S rDNA fiber, and one chromatid of the B chromosome’s long arm was connected with the intercalary region in the long arm of chromosome 1. In Figure 2E, somatic associations between the A and B chromosomes are shown for the genotype from the population of Ramat haNadiv (2*n* = 14 + 3B). This population belongs to the group of central populations, which comprises plants that are highly enriched with Spelt1 and Spelt52 TRs [14,17] (Figure 1; Table 1). Extended ectopic fibers connect the short arm of the B chromosome with the intercalary regions of two regular A chromosomes, and one of the fibers consists of 5S rDNA. Thus, across the different plants that we examined from the various populations, we found cell-specific rearrangements of B chromosomes and ectopic associations between B and regular A chromosomes in somatic tissues.

#### 2.1.2. Ectopic B-A Chromosome Associations and Rearrangements of B Chromosomes in Meiotic Cells

In the plants from different populations, cell-specific ectopic A-A and B-A chromosomal associations and B chromosome rearrangements were identified during meiosis I stages. In different genotypes, among 15 to 30 well-spread meiotic chromosome plates, analyzed on the same cytological slide, in most cases, ectopic associations between B and A chromosomes were detected in a cell-specific manner. The quantitative assessment of the cell-specific B-A chromosomal associations was conducted at the diplotene-anaphase I stages, facilitating proper chromosome identification. However, a large number of cells were ignored because failure to accurately discriminate ectopic associations and chromosome overlapping. Therefore, the real amount of cell-specific events was underestimated.

**Figure 3:**
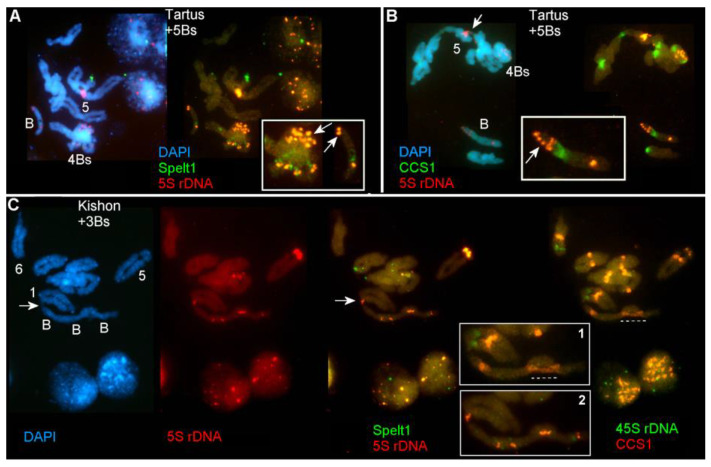

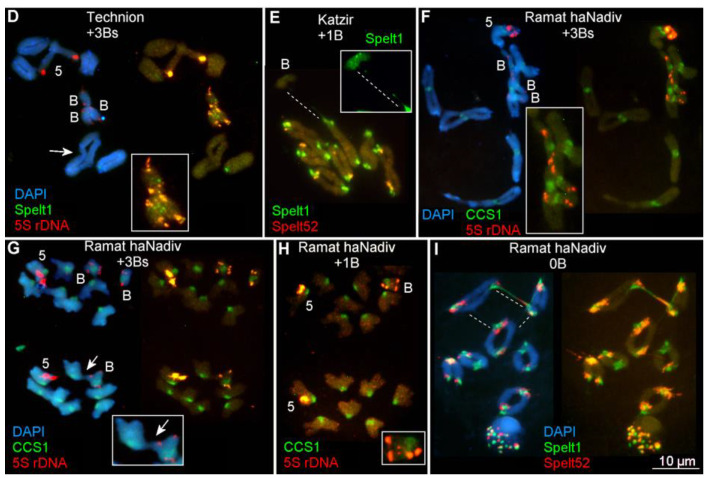
Ectopic chromosome associations and B chromosome rearrangements during microsporogenesis in different genotypes of *Aegilops speltoides*. Meiotic chromosomes at the diakinesis (**A**) and diplotene (**B**) stages of the plant from Tartus (2*n* = 2x = 14 + 5B). (**A**) Four B chromosomes form a tetravalent; in their short arms, the number of 5S rDNA clusters is doubled. The fifth B chromosome acts as a univalent; there is a single 5S rDNA cluster in its short arm (arrows, enlargement in the small box). (**B**) In the diplotene stage, four Bs form a tetravalent. In the univalent B, translocation of the 5S rDNA cluster occurs in the short arm (arrow, enlargement in the small box). Nonhomologous association of A chromosomes is indicated with arrow. (**C**) Meiotic chromosomes at the diakinesis stages of the plant from Kishon (2*n* = 2x = 14 + 3B). FISH with 5S rDNA (in red) and Spelt1 (in green); re-probing with 45S rDNA (in green) and CCS1 (in red). The three Bs synapse at their terminal regions and form a chain, which is associated ectopically with chromosome 1 (arrow) (enlargement in the small box 2). The pericentromeric regions of two Bs recombine (dashed line, small box 1). (**D**) Meiotic chromosomes at the diakinesis stage of the plant from Technion (2*n* = 2x = 14 + 3B). Three Bs form a trivalent (enlargement in the small box). Tetravalent of A chromosomes is shown with arrow. (**E**) Meiotic chromosomes at the diakinesis stage of the plant from Katzir (2*n* = 2x = 14 + B). Stretched Spelt1 fiber between the B and A chromosomes indicate ectopic recombination (dashed line, enlargement in the small box). (**F**) Meiotic chromosomes at the diakinesis stage of the plant from Ramat haNadiv (2*n* = 2x = 14 + 3B). Ectopic associations between three Bs and two bivalents of standard chromosomes are revealed (enlargement in the small box). (**G**) Meiotic chromosomes at the anaphase I stage of the plant from Ramat haNadiv (2*n* = 2x = 14 + 3B). Ectopic association between A and B chromosomes moving toward the same pool is stressed (arrow, enlargement in the small box). (**H**) Meiotic chromosomes at the anaphase I stage of the plant from Ramat haNadiv (2*n* = 2x = 14 + B). An additional 5S rDNA cluster is revealed in the long arm of the B chromosome (enlargement in the small box). (**I**) Meiotic chromosomes at the diakinesis stage of the plant from Ramat haNadiv (2*n* = 2x = 14). Ectopic associations between nonhomologous chromosomes are shown with dashed lines.

In the genotype Tartus (3B), among 30 cells analyzed on the same cytological slide, in 20 cases, B-A ectopic associations were detected. In the genotype Kishon (3B), among 28 cells analyzed, in 18 cells, B-A associations were revealed. In the genotype Technion (3B), among 32 cells analyzed on two slides, in 23 cells, B-A associations were revealed. In the genotype Ramat haNadiv (1B), among 15 cells analyzed on the same slide, in 9 cases, B-A associations were detected. In the genotype Ramat haNadiv (3B), among 23 cells analyzed on the same slide, in 19 cells, B-A associations were revealed. Relevant examples are shown in Figure 3.

In Figure 3A, meiotic chromosomes at the diakinesis stage of the genotype from the population of Tartus (2*n* = 2x = 14 + 5B) are shown. Here, four Bs form tetravalent, and the fifth B chromosome performs as a univalent. The univalent B has one 5S rDNA cluster in its short arm. In the tetravalent, the number of 5S rDNA clusters in the short arms is doubled that is the genotype-specific feature.

In Figure 3B, meiotic chromosomes at the diplotene stage of the same plant are shown. Four Bs form a tetravalent. In the short arm of the univalent B, a large translocated fragment containing a 5S rDNA cluster is detected; this is, probably, a consequence of recombination between Bs. Additionally, non-homologous ectopic association of chromosome 5 with another bivalent was found in this cell.

In Figure 3C, meiotic chromosomes of the genotype carrying three Bs (2*n* = 2x = 14 + 3B) from the marginal Kishon population [14,17] (Figure 1; Table 1) are presented. The three Bs form a chain via linkage of their terminal regions, and this chain, in turn, is ectopically linked with chromosome 1. Additionally, associations of 5S rDNA clusters and centromeres are revealed in Bs.

The plant from the marginal population of Technion [14,17] (Figure 1; Table 1), which possess three Bs (2*n* = 2x = 14 + 3B), is presented in Figure 3D. A single pair of A chromosomes carries a large Spelt1 cluster, and typical small intercalary Spelt1 clusters are found in all Bs. The three Bs form a trivalent via synapsis of two Bs along their entire length and connection with the terminal region of the third B. In this genotype, non-homologous A chromosomes form a tetravalent in most of the cells; this is indicative of genotype-specific rearrangement.

In Figure 3E, meiotic chromosomes of the plant (2*n* = 2x = 14 + B) from the intermediate population of Katzir (Figure 1; Table 1) are shown. Here, the stretched ectopic Spelt1 fiber between chromosomes B and A is emphasized.

Meiotic chromosomes of plants bearing zero to three Bs from the Ramat haNadiv population (Figure 1; Table 1) are presented in Figure 3F–I. Multivalent consisting of three Bs and chromosome 5 is emphasized in Figure 3F. Associations between the A and B chromosomes at anaphase I are shown in Figure 3G. In Figure 3H, meiotic cell at anaphase I is presented. In this plant, appearance of the second intercalary 5S rDNA cluster in the B chromosome long arm was detected in all the cells; that is, this is a genotype-specific feature. In Figure 3I, the genotype with the standard chromosome set is shown: here, non-homologous associations between distal-terminal chromosomal regions enriched with tandem repeats are stressed. 

Thus, ectopic B–A and A–A chromosome associations, and cell-specific and genotype-specific rearrangements of the B chromosomes were documented during microsporogenesis in plants from all the studied populations. 

### 2.2. Variability in the Copy Numbers of the Angela, Wilma, and Stasy Retrotransposons and Spelt1 Tandem Repeat in the Spike Tissues of Individual Plants

#### 2.2.1. Differences Between Individual Genotypes in Abundances of the *Angela*, *Wilma*, and *Stasy* Retrotransposons and Spelt1 Tandem Repeat

The copy numbers of the *Angela*, *Wilma*, and *Stasy* TEs and the Spelt1 TR were estimated in four types of spike tissues, specifically, pistils, pre-meiotic anthers, anthers in the meiosis I stages, and anthers in the meiosis II-young free pollen grain stages in six plants from the populations of Kishon (Figure 3C), Technion (Figure 3D), and Ramat haNadiv (Figure 3F,G). From each population, two plants, one with three Bs (2*n* = 2x = 14 + 3B) and the other without B chromosomes (2*n* = 2x = 14), were analyzed.

**Figure 4:**
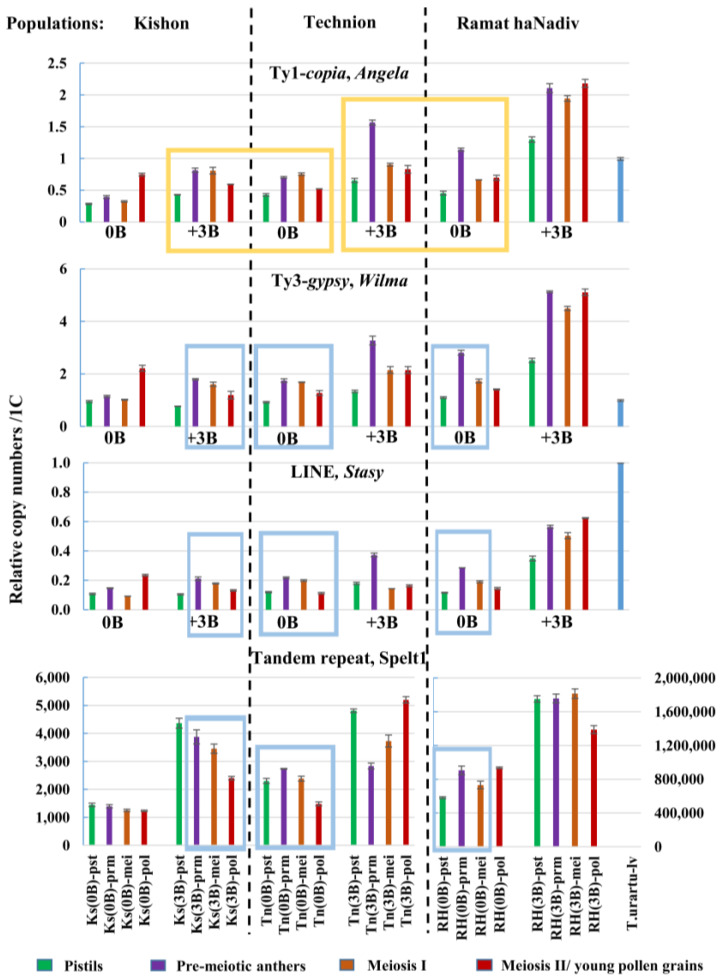
Relative copy numbers of the *Angela*, *Wilma*, and *Stasy* TEs and the Spelt1 TR per haploid genome in somatic and generative tissues of the spike in individual genotypes of *Ae. speltoides*. The genotypes (0B) and (3B), which are similar in the copy number dynamics for *Angela*, *Wilma* and *Stasy*, are indicated by yellow boxes. The genotypes Ks(0B) and RH(3B) differ in their TE contents, but they have similar copy number dynamics for *Angela*, *Wilma*, and *Stasy*. Spike tissues of the genotypes, which have similar copy number dynamics of Spelt1 and the three TEs, are indicated by blue boxes. The relative copy numbers are indicated along the vertical coordinate axis. Abbreviations for the genotypes are indicated along the horizontal axis. Genotypes from the Kishon population: Ks(0B), genotype without Bs; Ks(3B), genotype with three Bs. Genotypes from the Technion population: Tn(0B), genotype without Bs; Tn(3B), genotype with three Bs. Genotypes from the Ramat haNadiv population: RH(0B), genotype without Bs; RH(3B), genotype with three Bs. Abbreviations: pst, pistils; prm, pre-meiotic anthers; mei, anthers in meiosis I stages; pol, anthers in meiosis II/young free pollen grain stages; lv, leaves (for *T. urartu*).

Ty1-*copia*, *Angela*. The meiotic anthers in genotype Ramat haNadiv (3B) and the pistils in genotype Kishon (0B) had the highest 4,686 and lowest 697 number of copies of *Angela* per haploid genome, respectively (Figure 4, Table 2).

In plants carrying three Bs from the Kishon and Technion populations, the average copy number per genome of *Angela* was 1.5-fold higher than that in plants of genotype (0B) from the same population, and there was a 2.5-fold difference between the two genotypes from Ramat haNadiv.

Ty3-*gypsy*, *Wilma*. The copy number of *Wilma* ranged from 172 copies in the pistils in Kishon (0B) to 856 copies in the pre-meiotic anthers in Ramat haNadiv (3B) (Figure 4, Table 2). Two genotypes from the population of Kishon are similar in their average copy number of *Wilma*. In the genotypes with three Bs from Technion and Ramat haNadiv, the copy numbers are 1.5- and 2.5-fold higher than those in plants with zero Bs in their genome, respectively. 

LINE, *Stasy.* The retroelement *Stasy* was revealed in small copy numbers, specifically, from a minimum of 5 copies in different tissues of genotypes Kishon (0B), Kishon (3B), and Technion (0B) to a maximum of 32 copies in the meiotic anthers of Ramat haNadiv (3B) (Figure 4, Table 2). In the Kishon population, the average copy number of *Stasy* in the plants with and without Bs is similar. In plants carrying three Bs from Technion and Ramat haNadiv, the copy numbers are 1.3- and 2.5-fold higher, respectively, than the copy numbers in normal genotypes. 

Tandem repeat, Spelt1. Considerable differences were found between the six plants with regard to the genomic abundance of Spelt1. The copy number ranged from 173 in the meiotic anthers of the genotype Kishon (0B) to 190,825 copies in the meiotic anthers of the genotype Ramat haNadiv (3B): that is, there was almost a 950-fold difference in the lowest and highest copy number. In the Ramat haNadiv (3B), Technion (3B), and Kishon (3B) genotypes, the average Spelt1 copy number was twice that in the corresponding (0B) genotypes (Figure 4, Table 2).

Thus, the genomic abundances of the TEs and TR in plants with three Bs was higher than that in the plants with a normal set of chromosomes from the same population. The biggest difference were detected between two plants from the Ramat haNadiv population. 

#### 2.2.2. Differences in the Copy Numbers of the *Angela*, *Wilma*, and *Stasy* Retrotransposons and Spelt1 Tandem Repeat between Somatic and Generative Tissues of the Same Spike

In all six plants, the smallest copy number of all three TEs was found in the pistils. A sharp increase was observed in the pre-meiotic anthers, especially in plants with three Bs. For example, in genotype Technion (3B), a 2-fold difference between pistils and pre-meiotic anthers was observed for *Angela*, and for *Wilma* and *Stasy*, a 2.4-fold difference was observed. In meiotic anthers in the meiosis I stages, a significant decrease was observed in the copy numbers: specifically, a 1.6-fold drop was observed for *Angela*, a 1.5-fold drop for *Wilma*, and a 2.8-fold drop for *Stasy* (Figure 4, Table 2). Further, in anthers in the meiosis II-young pollen grain stages, an increase in the copy number for the three TEs was found only in two genotypes, that is, Kishon (0B) and Ramat haNadiv (3B), while the TE copy numbers decreased in the remaining four plants. 

The copy numbers of Spelt1 in the same tissues varied significantly between the 0B and 3B genotypes of the same population, and between different populations. There was a 2.9-fold difference between the Spelt1 copy numbers in pistils of the genotypes Kishon (0B) and Kishon (3B), a 2-fold difference between Technion (0B) and Technion (3B), and a 2.9-fold difference between Ramat haNadiv (0B) and Ramat haNadiv (3B). Further, the lowest and highest Spelt1 copy numbers were revealed in all spike tissues of the genotypes Kishon (0B) and Ramat haNadiv (3B), respectively (Figure 4, Table 2). Thus, in general, the copy numbers of TEs and TR seem to be greater in spike tissues in plants with three Bs than in plants without Bs from the same population. 

#### 2.2.3. Inter-Individual and Intra-Organismal Similarities in the Copy Number Dynamics of the *Angela*, *Wilma*, and *Stasy* retrotransposons and Spelt1 Tandem Repeat

Despite the differences in the abundances of the TEs and TR in the six plants (Figure 4, Table 2), a high positive correlation, *r* = 0.81 – 1.00, in the copy numbers of *Angela*, *Wilma*, and *Stasy* in four spike tissues of individual genotypes was revealed (Table 3). The Spelt1 tandem repeat demonstrated different from the TE copy number dynamics in the genotypes Kishon (0B), Technion (3B), and Ramat haNadiv (3B), *r* = −0.87–−0.38. 

However, in all spike tissues of genotype Technion (0B), the dynamics of Spelt1 was similar to that of the three TEs, *r* = 0.50–0.80 (Figure 4, blue boxes; Table 3). Despite low correlation in four tissues (Table 3), in the genotype Kishon (3B), the copy numbers of TEs and Spelt1 showed similar dynamics in pre-meiotic anthers, meiotic anthers in meiosis I stages, and meiotic anthers in meiosis II-young pollen grain stages, that is, in all tissues except for the pistils (Figure 4, blue boxes). Additionally, in the genotype Ramat haNadiv (0B), the tissue-specific dynamics of the three TEs and TR were similar in the pistils, pre-meiotic anthers, and anthers in the meiosis I stages (Figure 4, blue boxes). 

A high similarity in the abundance and/or intra-organismal dynamics of TEs was observed between plants with and without Bs from different populations. Specifically, a high degree of similarity was observed between genotype Kishon (3B) and Technion (0B), and between genotypes Technion (3B) and Ramat haNadiv (0B) (Figure 4, yellow boxes). Besides, despite the differences in the genomic abundances of the TEs and TR, the intra-organismal dynamics of the three TEs was similar in four spike tissues in the genotypes Kishon (0B) and Ramat haNadiv (3B) (Figure 4).

Hence, intra-organismal correlations were found with regard to the copy number dynamics of *Angela*, *Wilma*, and *Stasy* in all plants. Additionally, the Spelt1 dynamics was found to correlate with the TE dynamics in one of the 0B genotypes, and to partially correlate with the TE dynamics in the two other genotypes, 0B and 3B. Finally, genotypes 0B and 3B from different populations are similar in the genomic abundances and/or intra-organismal copy number dynamics of the TEs and TR.

## 3. Discussion

In this research, we focused on the cytogenetic features of Bs and the intra-organismal copy number dynamics of TEs and TR in plants with and without Bs in highly heterogeneous populations of *Ae. speltoides*.

### 3.1. Rearrangements and Ectopic Recombination in Both Mitosis and Meiosis as Inherent Characteristics of B Chromosomes

An essential feature of *Ae. speltoides* is a wide range of naturally occurring genotype- and cell-specific chromosomal rearrangements in the background of homologous and ectopic recombination [20,23,24]. In *Ae. speltoides*, Bs demonstrate a well-recognizable morphology and patterns of repetitive DNA. Somatic rearrangements of Bs, specifically, lack of the 5S rDNA cluster in the short arm and shortening of the distal part in the long arm [17,19], and ectopic B-A associations in microgametogenesis [20], have been previously reported. Here, we documented rearrangements of Bs in somatic cells in plants carrying 1 to 6 Bs in their genomes. The studied populations vary greatly in the patterns of Spelt1 and Spelt52 TRs, which to a large extent, determine the composition of distal/subterminal heterochromatin clusters [17,20] (Figure 2). In the present research, we were able to identify a limited spectrum of B-specific rearrangements with the help of three conservative chromosomal markers, namely, 5S rDNA, Spelt1 and TTTAGGG telomere repeat. In all the studied genotypes, repatterning of these clusters uncovered somatic recombination in individual cells. It is possible that a majority of the rearrangements occurs between Bs; however, in some cases, intrachromosomal rearrangements were obvious (Figure 2C). Furthermore, numerous ectopic associations with standard chromosomes point to A-B interchromosomal somatic exchanges. 

Somatic recombination is associated with DNA replication and repair processes and is tightly interlinked with individual chromosome positioning and dynamics in interphase nuclei [25,26,27,28]. During cell proliferation and differentiation, numerous endo- and exogenous factors cause DNA lesions, which are correctly repaired through comprehensive evolutionarily conserved mechanisms, or, when erroneously repaired or left unrepaired, lead to chromosomal aberrations, causing genome instability and, ultimately, cell death [29,30,31,32,33]. Ectopic homologous recombination occurs randomly and rarely [34,35], and has been observed between even spatially distant regions in somatic interphase nuclei in *Arabidopsis* [36]. In *Ae. speltoides*, homologous associations appear in 2% to 3% of the cells in genotypes with standard chromosome sets, while nonhomologous ectopic associations are found in the vast majority of cells, if not all, that is, in 100% of interphase nuclei [24]. Further, ectopic chromosome associations are found in 65.6% of cells in individual plant with a standard chromosome set [24]. Since homologous chromosomes are separated in the nuclear space, nonhomologous chromosomes serve as a source of non-allelic DNA repair via template switching [37,38] in the somatic interphase, and all types of repetitive sequences, especially transposable elements [21,39], appear to be the trigger for illegitimate recombination and expansion of repetitive sequences in the genome [40,41,42]. Hence, we predicted that Bs would participate in ectopic events, and indeed, we identified not only B–B, but also somatic associations of B and A chromosomes, in all genotypes. Ectopic recombination, which causes chromosomal rearrangements, may also prevent harmful aberrations, and, above all, double-stranded DNA breaks (DSBs), and therefore, it is vitally important for the cell [20,24,26,32]. In this regard, Bs serve as an additional template in DNA repair, especially in heterochromatin and clusters of various repetitive sequences, specifically, various transposable elements that are distributed throughout the euchromatin [21]. Vice versa, A chromosomes contribute to ectopic DNA repair in Bs, and contribute to the accumulation of sequences of standard chromosomes in Bs during both somatic and meiotic cell proliferation [43]. 

In meiosis, inter-chromosomal exchanges are mainly consequences of homologous recombination, which requires programmed DSB induction and repair [33,44]. However, the involvement of ectopic repetitive sequences in the repair of DNA damage is a complementary process both in plant somatogenesis and microgametogenesis [26,45]. A single B chromosome in the *Ae. speltoides* genome is responsible for 45% to 55% of B-A ectopic associations, whereas two B chromosomes mainly form a bivalent that is ectopically connected to A chromosomes in 19% of the cells in diakinesis [20]. 

Illegitimate associations are traced in the meiotic prophase II, and broken ectopic DNA strands are left unrepaired in haploid cells entering pollen mitosis I. Thus, both somatic and meiotic cells enter the next round of cell cycle with unrepaired and improperly condensed/decondensed DNA regions, which lead to chromosomal aberrations. In this way, chromosomal aberrations are transmitted from pre-meiotic cells to meiotic prophase I, and in addition to the consequences of meiotic recombination, it may cause numerous nonhomologous/ectopic recombination events that can be traced till the end of meiosis [20,24]. An odd number of Bs or a single B leads to an increase in the proportion of B-A ectopic associations and may affect the segregation of Bs in the meiotic (Figure 3G) and mitotic anaphase. In rye, synapsis and formation of the synaptonemal complex (SC) of B chromosomes take place in prophase I; when an odd number of Bs are present, SC formation may be impaired [46]. In addition to regular bivalent and normal SC formation, Bs form multivalent due to segmental synapsis, and univalent B performs intrachromosomal synapsis [46,47,48,49]. In *Ae. speltoides*, the synaptic configurations vary [15] (Figure 3), and alignments along entire lengths and/or terminal associations of Bs are observed in different genotypes and different cells. The B chromosome rearrangements, which have been revealed in meiosis, may be consequences of B–B, B-A, and intra-B-chromosomal rearrangements. Evidently, both in mitosis and meiosis, the selection in favor of maintaining a conserved B chromosome structure takes place at the intra-organismal and intra-population levels; however, the fixing of some rearrangements explains the current intraspecific polymorphisms observed in the morphology of Bs and repetitive DNA patterns [16,17,19].

### 3.2. Origin of B Chromosomes in Ae. speltoides

A specific characteristic of Bs in *Ae. speltoides* is the presence of the 5S rDNA cluster in both arms. Chromosome 5 exclusively harbors the main genomic cluster of 5S RNA genes. It can be assumed that accessory Bs arise as a result of the recombination of chromosome 5 with nonhomologous chromosome(s) [17]. Alternatively, Bs could also be the result of rearrangements of chromosome 5; that is, their origin might be monochromosomal. In different genotypes from different populations, various types of rearrangements in homo- and heterozygotes, such as para- and pericentric inversions, deletions, and translocations, have been previously reported for chromosome 5 [14,17], [20]. In addition, chromosome 5 is the source of supplementary mobile 5S rDNA clusters in the genome [23,50,51,52]. In any case, the similar sequence composition in the pericentromeric regions of As and Bs indicate an intraspecific origin of Bs in *Ae. speltoides* [16]. Stabilization of the arisen *de novo* B chromosome should be achieved through self-pollination insofar as *Ae. speltoides* is a rare species that exhibits dualism in its mating system, and transits from outcrossing to selfing in stressful environments [13,14]. Therefore, it is within reason to assume that the appearance of Bs in the genome of *Ae. speltoides* is an example of nonrandomness and canalization of chromosomal rearrangements. 

### 3.3. Role of B Chromosomes in Stabilizing the Genome

We previously showed that between the native plants and artificial intraspecific hybrids of *Ae. speltoides*, there were 2- to 4-fold fluctuations in the copy numbers of the Ty1-*copia*, Ty3-*gypsy*, and LINE TEs between genotypes, and there were 18- to 440-fold differences in the copy numbers of the species-specific Spelt1 TR between vegetative and generative tissues of the same plant [53]. Wide-ranging fluctuations in the TE and TR copy numbers were accompanied by extensive chromosomal rearrangements in both the parental and hybrid genotypes. 

In the present study, we found that there were similar fluctuations in the TE and TR copy numbers in native plants with normal chromosome sets and with three Bs in their genomes. Although B chromosomes are not a vital element of the genome, their presence undoubtedly affects its stability and functioning. However, we did not find an undeniable influence of Bs on the total genomic abundances and dynamics of repetitive sequences. The presence of B chromosomes causes an increase in the proportion of various repetitive DNA sequences, as each B adds up to 10% to the genome size in *Ae. speltoides* [19]. Here, we observed an increase in the genomic content of TEs in plants with three Bs as compared with plants without Bs from the same population, but this may also mirror wide inter-individual polymorphisms. Further, the dynamics in the copy numbers of the three TEs were similar in all the investigated plants. 

The low abundance of Spelt 1 in the genome of plants from Kishon and Technion is determined by the presence of only one or two subterminal clusters of medium size and small intercalary clusters on chromosome 4 and Bs, while plants from the Ramat haNadiv population contain hundreds of more copies of Spelt1. In the genomes Kishon (3B) and Technion (3B), Bs can indeed make a visible contribution to the overall low content of Spelt1 and, probably, to its copy number dynamics. However, in plants from Ramat haNadiv, which have a high genomic content of Spelt1, it is unlikely that Bs play any significant role in the TR abundances and intra- and inter-individual copy number fluctuations. The almost 2-fold difference in the total Spelt1 copy number between Ramat haNadiv (0B) and Ramat haNadiv (3B), rather, mirror a high frequency of recombination in the heterochromatin, which leads to depletion or amplification of repetitive sequences within chromosomal clusters without causing a change in the number of clusters. This finding has been previously reported for the Spelt52 TR in *Ae. speltoides* [52]. Thus, chromosome patterns may appear similar in plants from the same population, while the number of copies of TR is different.

Regardless of the presence or absence of Bs, intra-organismal copy number fluctuations in TEs and TR were found highly similar in the contrasting genotypes Kishon (3B), Ramat haNadiv (0B), and Technion (0B) (Figure 4). These observed dynamics point to the co-localization of TEs and TR in heterochromatic blocks, which are subject to recombination as a whole unit. 

Our data show that Bs do not affect the intraorganismal dynamics of *Angela*, *Wilma*, *Stasy*, and Spelt1 sequences, and probably do not have any significant impact on the inter-individual variability in the copy numbers of the TEs and TR. This may be attributable to the low abundances of these sequences in Bs. Specifically, plants Kishon (3B) and Technion (0B) are almost similar both in TE abundances and dynamics (Figure 4, yellow boxes). In contrast, the genotypes Kishon (0B) and Ramat haNadiv (3B) show variability in their TE copy numbers, while the intraorganismal TE dynamics are similar. We observed that the genomic content of the three TEs increases from genotype Kishon (0B) to genotype Ramat haNadiv (3B) as the number of Spelt1 copies in the genome increases (Figure 4; Table 2). This observation, along with the similarity in the copy number dynamics, indicates that TEs and TR are part of common clusters. In the Bs, this is probably in the form of a small intercalary cluster that is marked by the presence of Spelt1 in the long arm. This is a possible limitation of our study, as we have not elucidated the undeniable relationship between Bs and the copy number dynamics of the studied TEs and TR.

Fluctuations in the total TE and TR copy number between different tissues of one spike is another phenomenon that is indicative of the efficacy of DNA repair during cell proliferation and differentiation [53]. In the present study, we used real-time qPCR with specific primers to evaluate the number of copies of *Angela*, *Wilma*, *Stasy*, and Spelt1, therefore, any altered sequences in the primers’ annealing sites were not counted. In maize, the production of reactive oxygen species as a major source of DNA damage is reduced in floral stem cells due to naturally created hypoxic conditions [54,55]. Evidently, the proportion of altered DNA sequences is lower in pre-meiotic anthers than in leaves and pistils. In archesporial cells, reprogramming to gametogenesis triggers the expression of meiosis-specific genes and is associated with accumulation of specific meiotic gene transcripts, including those of genes that control the meiotic cell cycle and those responsible for DNA replication, DSB formation and repair, chromosome pairing, synapsis, recombination, meiotic chromosome condensation, and so on [55,56]. Presumably, in a hypoxic environment, in pre-meiotic anthers, DNA damage occurs to a lesser extent and the efficacy of DNA repair is significantly higher than that in other somatic tissues. In meiosis I, both homologous and ectopic recombination can cause an increase in the number of DNA lesions, and this depends on the type and rate of chromosomal aberrations in individual genotypes. 

In qPCR analysis, DNA lesions in somatic tissues contribute to the final total number of intact amplicons. In anthers in the meiosis II-young pollen grain stages, the impact of gradually degraded somatic tissues is minimized [57], and the DNA sample contains mainly haploid meiocytes, which, in turn, undergo preliminary intraorganismal selection against harmful DNA damage/chromosomal aberrations. As mentioned above, heterozygosity in chromosomal rearrangements and wide inter-individual polymorphism in repetitive DNA contents and patterns are the inherent features of *Ae. speltoides*. Hence, the copy numbers of intact TE and TR sequences is dependent on the genotype and the tissue type, and it is difficult to separate the influence of B chromosomes from that of other factors.

The data obtained in the present study indicate dynamic reorganization of the genome during ontogenesis, and there was no evident negative consequence of the presence of Bs. On the one hand, along with standard A chromosomes, supernumerary chromosomes, especially when present singly or in odd numbers, are involved in chromosomal rearrangements through ectopic recombination, and this is one of the mechanisms of non-allelic DNA repair that is crucial for the genome. Rearrangements of A chromosomes reduce or prevent normal meiotic synapsis and recombination, but seemingly, do not restrict ectopic recombination. On the other hand, Bs function, to a large extent, independently of As, and do not form SC and recombine with As [1,2,46]. Thus, their negative impact is limited due to their specific architecture, as we observed in this research, too. When Bs are present in small amounts, that is, one to three Bs to a genome, they do not significantly reduce male fertility and can even increase vigor, and such plants might have benefits under changing environmental conditions [14,15]. These benefits, in turn, may explain the preservation of B chromosomes in natural populations and selection in favor of the conservation of B-specific architecture. Eventually, the maintenance of Bs in the population and their specific organization and behavior are under genomic control and are closely associated with intraspecific diversification and, ultimately, with the evolution of *Ae. speltoides*.

## 4. Materials and Methods 

### 4.1. Plant Material

Native *Ae. speltoides* plants carrying one to six B chromosomes and plants without Bs in the genome from contrasting allopatric populations [17], specifically, from the marginal populations of Kishon (Israel), Technion (Israel), and Tartus (Syria), the population of the intermediate type of Katzir (Israel), and the central population of Ramat haNadiv (Israel) (Table 1; Figure 1), were analyzed using FISH and real-time qPCR approaches. 

### 4.2. Fluorescence In Situ Hybridization Experiments

For FISH experiments, cytological slides of individual apical shoot meristems and individual anthers containing well-spread chromosomal plates were used. Chromosome spreading, DNA probes labeling, and FISH procedures were conducted as previously described [53]. The tandem repeats Spelt1 [58], Spelt52 [59], and pSc119.2 [60], pTa71 (for localization of 45S rDNA) [61], As5SDNAE (for localization of 5S rDNA) [62], CCS1 (cereal centromere-specific sequence 1) [63], and a PCR-generated synthetic probe consisting of a tandem repeat array of the short sequence TTTAGGG for the localization of telomeres [64] were used as DNA probes for FISH. The DNA probes were directly labeled with Cy-3 and fluorescein-12-dUTP (Jena Bioscience, Jena, Thuringia, Germany) using a standard protocol. The chromosomes were differentially stained with 4′,6-diamidino-2-phenylindole (DAPI). The slides were examined on a Leica DMR microscope (Leica Microsystems Wetzlar GmbH, Wetzlar, Germany) equipped with a Leica DFC300 FX CCD camera.

### 4.3. DNA Isolation and Cytogenetic Screening

Genomic DNA (gDNA) was isolated from plants from populations of Kishon, Technion, and Ramat haNadiv. Two plants were selected from each population—one with a normal set of chromosomes (2*n* = 2x = 14) and the other with three Bs in the genome (2*n* = 2x = 14 + 3B). The genotype abbreviations are as follows: plants from the Kishon population: Ks(0B) (2*n* = 2x = 14) and Ks(3B) (2*n* = 2x = 14 + 3B), plants from the Technion population: Tn(0B) (2*n* = 2x = 14) and Tn(3B) (2*n* = 2x = 14 + 3B), plants from the Ramat haNadiv population: RH(0B) (2*n* = 2x = 14) and RH(3B) (2*n* = 2x = 14 + 3B). 

Young spikes in the microsporogenesis stage were used for meiotic chromosome analysis and gDNA extraction from the pistils and anthers, and these procedures were performed as previously described [53]. Specifically, gDNA was extracted using the NucleoSpin Plant II kit (Macherey-Nagel GmbH & Co. KG, Düren, Germany). In brief, the young spikes in the microsporogenesis stage were collected in a fixative (a 3:1 ratio of absolute ethanol to glacial acetic acid) and stored at +4 °C until further analysis. The anthers in the different stages of development, specifically, (1) young pre-meiotic anthers with somatically dividing cells, (2) anthers in meiosis I (prophase I to anaphase I stages), and (3) anthers in meiosis II (dyads/tetrads to immature free pollen grain stages), were collected separately for gDNA extraction and FISH. The meiotic stages were precisely determined by microscopic examination of one individual anther, and two anthers from the same flower were collected for the following procedures. (4) Due to the small amount of tissue available for analysis, the pistils were collected from all flowers at different stages of development. The DNA concentration and purity were assessed using a NanoDrop^™^ Spectrophotometer 1000 (Thermo Scientific, Wilmington, DE, USA). The DNA from all the samples was of similar purity and quality.

### 4.4. Real-Time Quantitative PCR, Retrotransposon Sequence Sources, and Primer Design

The relative and absolute copy numbers of the Ty1-*copia* (*Angela*), Ty3-*gypsy* (*Wilma)*, and LINE (*Stasy*) TEs and the species-specific TR Spelt1 were determined in the spike tissues of six individual genotypes. The relative and absolute copy numbers of the TEs and TR were determined in different spikes tissues, specifically, the (1) pistils, (2) anthers with pre-meiotic (archesporial) cells, (3) anthers in the meiosis I stage, and (4) anthers in the meiosis II/ immature free pollen grain stage. gDNA extracted from the young leaves of *Triticum urartu* Thum. et Gandil. (AA genome, 2*n* = 2x = 14) was analyzed as a control reference sample in each experiment. The complete sequences of TEs were obtained from the TREP (Triticeae REPeat) http://wheat.pw.usda.gov/ITMI/Repeats/) and NCBI (http://www.ncbi.nlm.nih.gov/) databases.

The primers for real-time qPCR were designed using the Primer Express 2.0 software (Applied Biosystems, Waltham, Massachusetts, USA). The primer sequences for the unique regions of the TEs and TR and the oligonucleotides used as standards (synthetic oligonucleotides corresponding to the expected PCR product for each target) were designed as reported previously [53].

The reactions were performed in a 20-µL reaction volume containing 10 µL of 2X Fast SYBR™ Green Master Mix (Applied Biosystems), 0.75 µL of the forward primer (10 µmol), 0.75 µL (10 µmol) of the reverse primer, 5 ng of genomic DNA as the template (1.0 ng/µL), and 3.5 µL of ultra-pure water. The qPCR reactions were analyzed using a StepOne™ Plus Real-Time PCR system and StepOne Software version 2.2.2 (Applied Biosystems). The following reaction parameters were used: 20 s at 95 °C, followed by 40 cycles at 95 °C for 3 s and 60 °C for 30 s. The PCR product melting curves were assessed to verify that there was a single product for each primer pair: 60–95 °C range; 0.3 °C step. Additionally, single qPCR products were validated using electrophoresis on 1.5% agarose gels. To examine whether the amplification of the standard oligonucleotides (used for standard curve construction) is affected by the presence/ absence of genomic DNA, control reactions were performed in the presence of mammalian genomic DNA from the mole rat *Spalax galili* (2n = 52, *Spalacidae, Rodentia*). No significant differences were observed between the two conditions. The efficacy of the PCR reactions was confirmed based on a correlation coefficient value of ~1 and a slope of −3.3 ± 0.1 of the standard curve. The reactions were performed in triplicate. In the control wells containing ultra-pure double-distilled water (instead of template DNA), no amplification was detected. The target copy numbers were calculated from standard curves generated by a serial ten-fold dilution of the synthetic oligonucleotides corresponding to the expected amplicon for each target. The calculated target quantities were normalized to the single-copy vernalization gene (*VRN1*) [65]. To allow combining data from different PCR runs, *T. urartu* was used as the reference (assigned as “1”). To obtain the absolute copy numbers per haploid genome [53], the number of copies of each target and reference *VRN1* gene in 1 ng of DNA was calculated by multiplying the mean quantities (obtained as pg for *Angela, Wilma*, and Spelt1, and as fg for *Stasy* and *VRN1*) by the number of molecules of each amplicon in 1 pg (picogram; 1 pg = 10^−12^ g) (for *Angela, Wilma*, and Spelt1) or 1 fg (femtogram; 1 fg = 10^−15^ g) (for *Stasy* and *VRN1*) of DNA. Subsequently, the copy numbers of the targets in 1 ng of DNA were divided by the copy numbers of *VRN1* in 1 ng of DNA. The relative quantification (RQ) of the three TEs and TR was conducted by ∆∆Ct method using the single-copy *VRN1* gene as a reference. To allow the comparison of the relative copy numbers between the different targets from different PCR runs, the *T. urartu* standard gDNA sample was also measured and assigned as “1” (∆Ct_Sample_ = Ct_Target_ − Ct_VRN1_; ∆∆Ct = ∆Ct_Sample_ − ∆Ct_Tu_; RQ = 2^−∆∆Ct^) [65,66]. 

## Figures and Tables

**Table 1:** Description of the populations of *Ae. speltoides*, the accession numbers and sources of plant material.

No.	Populations: Origin, Source	Geographical Zone, Elevation, and Coordinates	Population Size, Location	Morphotype
1	Tartus, Syria ^1^ PI 487238	Mediterranean 600 m 35°07′ N, 36°07′ E	N/A; cultivated field *	ssp. *aucheri*
2	Kishon, Israel ^2^ 2.22	Mediterranean 2 m 32°48′ N; 35°02′ E	small; natural habitat endangered	ssp. *ligustica* ssp. *aucheri*
3	Technion, Israel ^2^ 2.36	Mediterranean 265 m 32°46′ N, 35°00′ E	small; natural abitat; extinct	ssp. *ligustica*, ssp. *aucheri*
4	Ramat haNadiv, Israel ^2^ 2.46	Mediterranean 100–125 m 32°33′ N, 34°56′ E	big; natural habitat, interrupted area	ssp. *ligustica*, ssp. *aucheri*, *intermediate*
5	Katzir, Israel ^2^ 2.93	Mediterranean 233–250 m 32°29′ N, 35°05′ E	big; natural habitat	ssp. *aucheri*

Source: ^1^—USDA, United States Department of Agriculture; ^2^—IE, Institute of Evolution collection, Haifa, Israel; *—data obtained by Google Earth.

**Table 2:** Copy numbers in absolute quantification per haploid genome of *Angela*, *Wilma*, and *Stasy* retrotransposons and Spelt1 tandem repeat in spikes of *Ae. speltoides* individual genotypes.

	Ty1*-copia*, *Angela*	Ty3-*gypsy*, *Wilma*	LINE, *Stasy*	Tandem Repeat, Spelt1
**Genotype**	copies/1C	copies/1C	copies/1C	copies/1C
**Kishon (0B)**				
Ks(0B)-pst	697	172	6	204
Ks(0B)-prm	966	205	8	195
Ks(0B)-mei	796	182	5	175
Ks(0B)-pol	1,729	385	12	173
*average*	*1047*	*236*	*8*	*187*
*max/min ratio* *	*2.5*	*2.2*	*2.4*	*1.2*
**Kishon (3B)**				
Ks(3B)-pst	1,030	140	5	586
Ks(3B)-prm	1,847	315	11	521
Ks(3B)-mei	1,836	283	9	447
Ks(3B)-pol	1,381	213	7	329
*average*	*1524*	*238*	*8*	*471*
*max/min ratio* *	*1.8*	*2.3*	*2.2*	*1.8*
**Technion (0B)**				
Tn(0B)-pst	1,103	164	5	311
Tn(0B)-prm	1,531	310	14	377
Tn(0B)-mei	1,790	294	10	323
Tn(0B)-pol	1,303	225	5	204
*average*	*1432*	*248*	*8*	*304*
*max/min ratio* *	*1.6*	*1.9*	*2.8*	*1.8*
**Technion (3B)**				
Tn(3B)-pst	1,599	235	8	634
Tn(3B)-prm	3,377	558	20	383
Tn(3B)-mei	2,057	373	7	499
Tn(3B)-pol	1,880	334	8	689
*average*	*2228*	*375*	*11*	*551*
*max/min ratio* *	*2.1*	*2.4*	*2.9*	*1.8*
**Ramat haNadiv (0B)**				
RH(0B)-pst	1,107	197	6	64,533
RH(0B)-prm	2,419	486	17	99,552
RH(0B)-mei	1,575	301	9	79,481
RH(0B)-pol	1,615	250	7	101,945
*average*	*1679*	*309*	*10*	*86378*
*max/min ratio* *	*2.2*	*2.5*	*2.8*	*1.6*
**Ramat haNadiv (3B)**				
RH(3B)-pst	2,913	432	18	184,644
RH(3B)-prm	4,510	856	30	185,272
RH(3B)-mei	4,201	754	26	190,825
RH(3B)-pol	4,686	852	32	147,443
*average*	*4078*	*723*	*26*	*177046*
*max/min ratio* *	*1.6*	*2.0*	*1.8*	*1.3*
		***T. urartu***		
*T. urartu*-lv	3,192	713	27	1

* The ratio between the maximal and minimal TE copy numbers.

**Table 3:** Correlation between *Angela*, *Wilma*, *Stasy*, and Spelt1 relative copy numbers in spike somatic and meiotic tissues of *Ae. speltoides* individual genotypes.

Genotype		TE/TE and TE/TR Correlation		
		***Wilma***	*Stasy*	Spelt1
**Kishon (0B)**	*Angela*	1.00	0.97	−0.62
	*Wilma*		0.96	−0.62
	*Stasy*			−0.44
**Kishon (3B)**	*Angela*	0.99	0.96	−0.16
	*Wilma*		0.98	−0.15
	*Stasy*			0.02
**Technion (0B)**	*Angela*	0.97	0.91	0.50
	*Wilma*		0.90	0.45
	*Stasy*			0.80
**Technion (3B)**	*Angela*	0.97	0.92	−0.87
	*Wilma*		0.81	−0.79
	*Stasy*			−0.75
**Ramat haNadiv (0B)**	*Angela*	0.97	0.96	0.73
	*Wilma*		1.00	0.57
	*Stasy*			0.54
**Ramat haNadiv (3B)**	*Angela*	1.00	0.98	−0.44
	*Wilma*		0.97	−0.38
	*Stasy*			−0.60

## Abbreviations

A	A chromosome
B	B chromosome
DAPI	4′,6-diamidino-2-phenylindole
FISH	fluorescent in situ hybridization
qPCR	real-time quantitative PCR
TE	transposable element
TR	tandem repeat
